# Intravital Imaging of Vascular Transmigration by the Lyme Spirochete: Requirement for the Integrin Binding Residues of the *B*. *burgdorferi* P66 Protein

**DOI:** 10.1371/journal.ppat.1005333

**Published:** 2015-12-18

**Authors:** Devender Kumar, Laura C. Ristow, Meiqing Shi, Priyanka Mukherjee, Jennifer A. Caine, Woo-Yong Lee, Paul Kubes, Jenifer Coburn, George Chaconas

**Affiliations:** 1 Department of Biochemistry & Molecular Biology, University of Calgary, Calgary, Alberta, Canada; 2 Department of Microbiology, Immunology & Infectious Diseases, University of Calgary, Calgary, Alberta, Canada; 3 Calvin, Phoebe and Joan Snyder Institute for Chronic Diseases, University of Calgary, Calgary, Alberta, Canada; 4 Graduate Program in Microbiology, Immunology, and Molecular Genetics, Medical College of Wisconsin, Milwaukee, Wisconsin, United States of America; 5 Center for Infectious Disease Research, Medical College of Wisconsin, Milwaukee, Wisconsin, United States of America; 6 Department of Physiology & Pharmacology, University of Calgary, Calgary, Alberta, Canada; 7 Division of Infectious Diseases, Department of Medicine, Medical College of Wisconsin, Milwaukee, Wisconsin, Unites States of America; University of Montana, UNITED STATES

## Abstract

Vascular extravasation, a key step in systemic infection by hematogenous microbial pathogens, is poorly understood, but has been postulated to encompass features similar to vascular transmigration by leukocytes. The Lyme disease spirochete can cause a variety of clinical manifestations, including arthritis, upon hematogenous dissemination. This pathogen encodes numerous surface adhesive proteins (adhesins) that may promote extravasation, but none have yet been implicated in this process. In this work we report the novel use of intravital microscopy of the peripheral knee vasculature to study transmigration of the Lyme spirochete in living *Cd1d* ^*-/-*^mice. In the absence of iNKT cells, major immune modulators in the mouse joint, spirochetes that have extravasated into joint-proximal tissue remain in the local milieu and can be enumerated accurately. We show that BBK32, a fibronectin and glycosaminoglycan adhesin of *B*. *burgdorferi* involved in early steps of endothelial adhesion, is not required for extravasation from the peripheral knee vasculature. In contrast, almost no transmigration occurs in the absence of P66, an outer membrane protein that has porin and integrin adhesin functions. Importantly, P66 mutants specifically defective in integrin binding were incapable of promoting extravasation. P66 itself does not promote detectable microvascular interactions, suggesting that vascular adhesion of *B*. *burgdorferi* mediated by other adhesins, sets the stage for P66-integrin interactions leading to transmigration. Although integrin-binding proteins with diverse functions are encoded by a variety of bacterial pathogens, P66 is the first to have a documented and direct role in vascular transmigration. The emerging picture of vascular escape by the Lyme spirochete shows similarities, but distinct differences from leukocyte transmigration.

## Author Summary

Lyme disease is the most common vector-transmitted infection in North America and Europe. Diverse clinical manifestations of Lyme disease result from the dissemination of the spirochetes causing the disease into a variety of tissue sites. Dissemination results from invasion of the vasculature by the bacteria, followed by exit into virtually all tissue types. The mechanism of vascular transmigration by Lyme disease spirochetes remains uncharacterized. Here we describe a novel approach to study transmigration of Lyme disease spirochetes using intravital microscopy of the peripheral knee vasculature in living mice. Our studies have identified an adhesin, P66, and its integrin-binding function as playing important roles in Lyme spirochete transmigration and dissemination.

## Introduction

**Fig 1:**
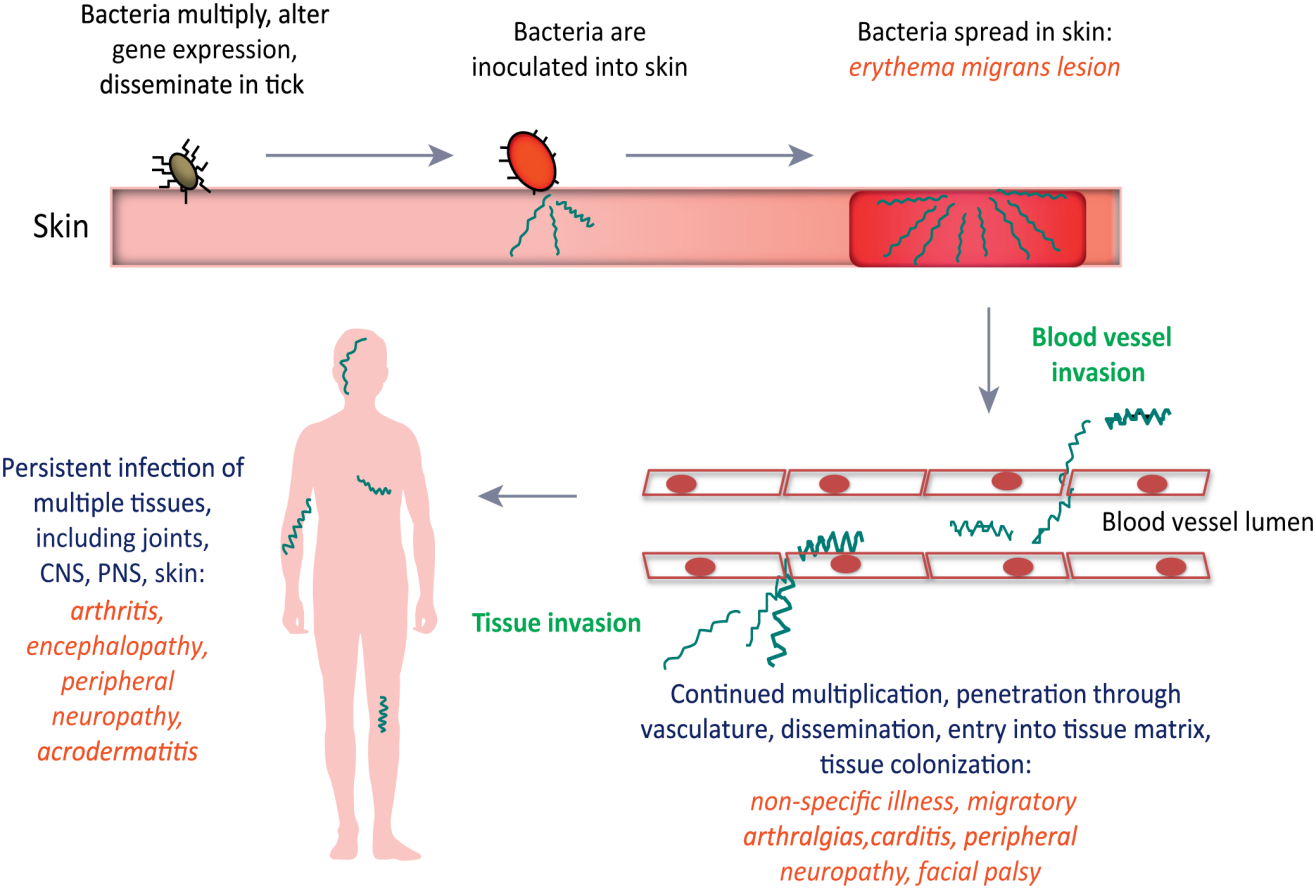
Progression of Lyme disease in humans following *B*. *burgdorferi* infection. Spirochetes are inoculated in the skin through the bite of an infected hard-shelled *Ixodes* tick. CNS, central nervous system; PNS, peripheral nervous system. Reprinted from Trends in Microbiology, Vol. 21, No. 8, Coburn, J., Leong, J. and Chaconas, G., Illuminating the roles of the *Borrelia burgdorferi* adhesins, Pages 372–379, Copyright 2013, with permission from Elsevier. [7]

Lyme disease is a spirochetal illness caused by various members of the genus *Borrelia*, and the most prevalent vector-borne illness in North America and Europe [1–5]. The disease is transmitted to humans during the feeding of infected hard-shelled ticks that have acquired the spirochetes during an earlier blood meal on infected reservoir animals. Once inoculation of the skin has occurred, the highly motile *Borrelia* species multiply and migrate in the skin, often resulting in an erythema migrans lesion or “bulls-eye rash”. As the disease progresses (see Fig 1), spirochetes invade the vasculature, which provides a mechanism for dissemination throughout the body, followed by extravasation into a variety of tissue types. Dissemination of spirochetes can result in non-specific illness, arthralgia, carditis and neuroborreliosis. Persistent untreated infection can result in acrodermatitis and a variety of neurological problems, as well as Lyme arthritis, a common feature of the disease in North America that results from the inflammatory response to spirochete invasion into the joints [6].

Although hematogenous dissemination is important for disease development, little is known regarding the mechanisms involved in this process. Hematogenous dissemination is a multi-step process likely involving multiple *Borrelia* proteins as well as host proteins and macromolecular cell receptors in the host vasculature [7, 8]. Direct analysis and quantification of the transmigration process has been complicated by a variety of factors. Generalized dissemination assays by culture of spirochetes from mouse tissues or by direct observation do not provide quantitative information [9]. More recently visualization of dissemination by whole body bioluminescent imaging [10] provides quantitative information. The above methods provide useful information, but do not reveal the causes for a lack of dissemination or the step along the transmigration pathway where defects occur. A failure to disseminate may be related to a lack of spirochete survival due to metabolic defects [11], clearance of spirochetes by the host immune system [12, 13], or a defect in one of the spirochetal components directly required for early or later steps along the transmigration pathway.

We have previously used intravital microscopy to study early events in dissemination [14–16]. The high dose needle inoculation and short-term observation period precludes the use of our intravital system for investigation of many aspects of the natural infectious cycle of *B*. *burgdorferi* in mice. However, it does provide an exceedingly powerful approach for investigation of mechanistic aspects of spirochete-host interactions in their natural setting. In particular, interactions of *B*. *burgdorferi* with the host microvasculature can be imaged in real time at high resolution under shear force in a living animal. This has allowed us to effectively image *B*. *burgdorferi* interactions with post-capillary venules [14–16]. Spirochetes in the vasculature have been observed to participate in initial tethering interactions, followed by longer-lived dragging interactions and finally stationary adherence to the endothelium. These interactions can be quantitatively analyzed and mechanistically dissected. However, invasion of surrounding tissue is a rare event compared with vascular adhesion and the number of transmigrating spirochetes observed by intravital microscopy was exceedingly low during the course of one-hour experiments. Moreover, transmigrated spirochetes were highly motile in flank skin, where the experiments were performed, and rapidly escaped the site of transmigration. Therefore, enumeration of transmigration required direct observation of rare escaping spirochetes, making the use of intravital microscopy ineffective for studying this process.

In the current work we report the development of a high resolution intravital imaging transmigration assay that allows enumeration of transmigrated spirochetes in the peripheral knee vasculature of living mice. In knee joint-proximal tissue in the mouse, iNKT cells surround the outside of blood vessels and block extravasation; they also eliminate those spirochetes that do successfully transmigrate into the joint-proximal tissue [17]. The use of *Cd1d* ^*-/-*^ mice, which lack iNKT cells, results in a 25-fold increase in spirochetes disseminating into the mouse joint [18], providing numbers of spirochetes amenable for direct visualization. Moreover, fluorescent transmigrated *B*. *burgdorferi* persist in the joint-proximal tissue and can be enumerated at 24 hours post intravenous (iv) inoculation. Using this new approach, coupled with an analysis of vascular clearance, we show that *B*. *burgdorferi* lacking BBK32, an important fibronectin and glycosaminoglycan adhesin [7, 19–21] display wild-type levels of transmigration. In contrast, spirochetes lacking the integrin adhesin and porin P66 [22–24] or those specifically defective in integrin binding display almost no vascular transmigration into the mouse knee joint-proximal tissue, indicating a direct role for P66-integrin interactions in the transmigration process.

## Results

### Monitoring transmigration of *B*. *burgdorferi* into knee joint-proximal tissue of *Cd1d* ^*-/-*^ mice

In our previous work we noted a 25-fold increase in spirochete burden in the knee joints of *Cd1d* ^*-/-*^ mice versus isogenic wild-type mice. More recently, our use of intravital microscopy of the peripheral knee vasculature of infected *Cd1d* ^*-/-*^ mice revealed readily observable spirochetes in the joint-proximal tissue [17], prompting us to further develop and exploit this system to study the transmigration process in the absence of spirochete killing by iNKT cells. The area exposed for intravital imaging in this study was over the patellar ligament and to the medial side of the right hind limb. We examined post-capillary venules that drain blood from the anterior tibialis muscle and join with the anterior tibial vein that drains into the great saphenous vein. The location of the venules ranges from 0 to a maximal depth of approximately 200 μm. These venules are located near the surface, rather than in the interior of the joint and transmigrating spirochetes are found predominantly in the anterior tibialis muscle. The venules display properties that are unique compared with venules in other tissue. The iNKT cells appear to be the first line of defense against *B*. *burgdorferi* in only the peripheral joint tissue; they have an extravascular location and are mobile compared to in the liver where they are intravascular and stationary [17, 18].

**Fig 2:**
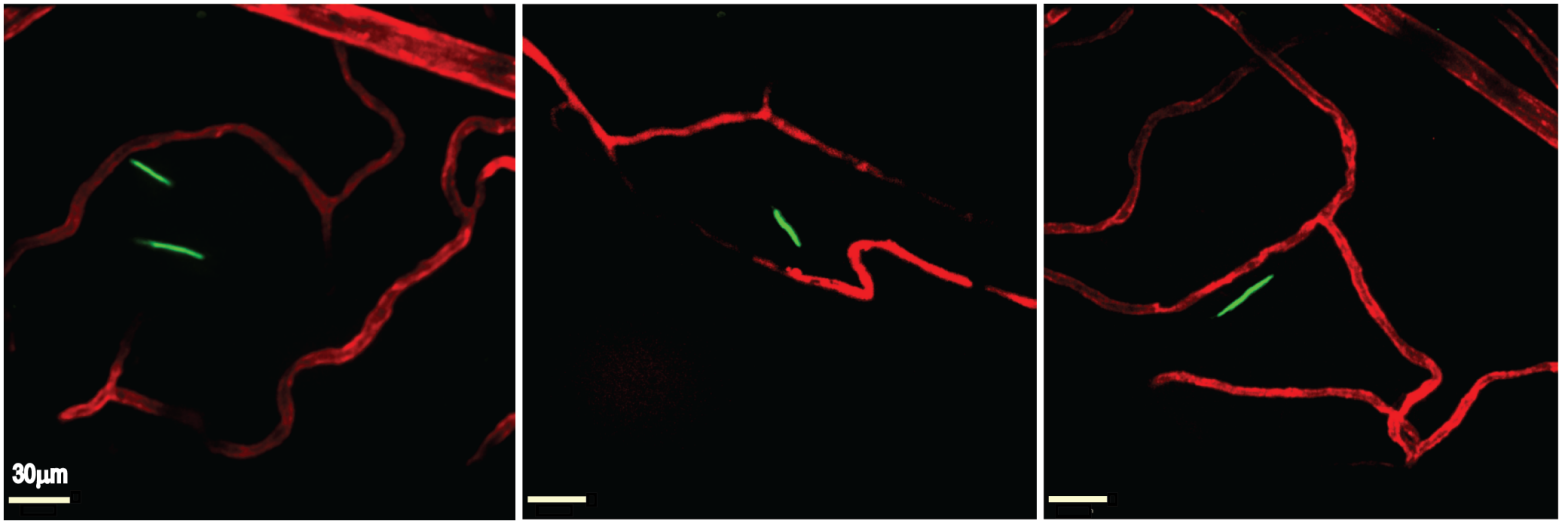
Intravital imaging of transmigrated *B*. *burgdorferi* in the knee joint-proximal tissue of a living mouse. Infectious GFP-expressing (green) *B*. *burgdorferi* (left and right panels GCB726, central panel GCB966, 4x10^8^ spirochetes per mouse) were injected into the tail vein of *Cd1d* ^*-/-*^ mice. At 20 hours post-inoculation, vascular transmigration was scored in the knee joint-proximal tissue of living mice by intravital microscopy using a spinning disk laser confocal microscope. Blood vessels were stained with Alexa Fluor 555-conjugated PECAM-1 antibody (red). Video footage of transmigrated spirochetes is shown in S1 and S2 Videos.

**Fig 3:**
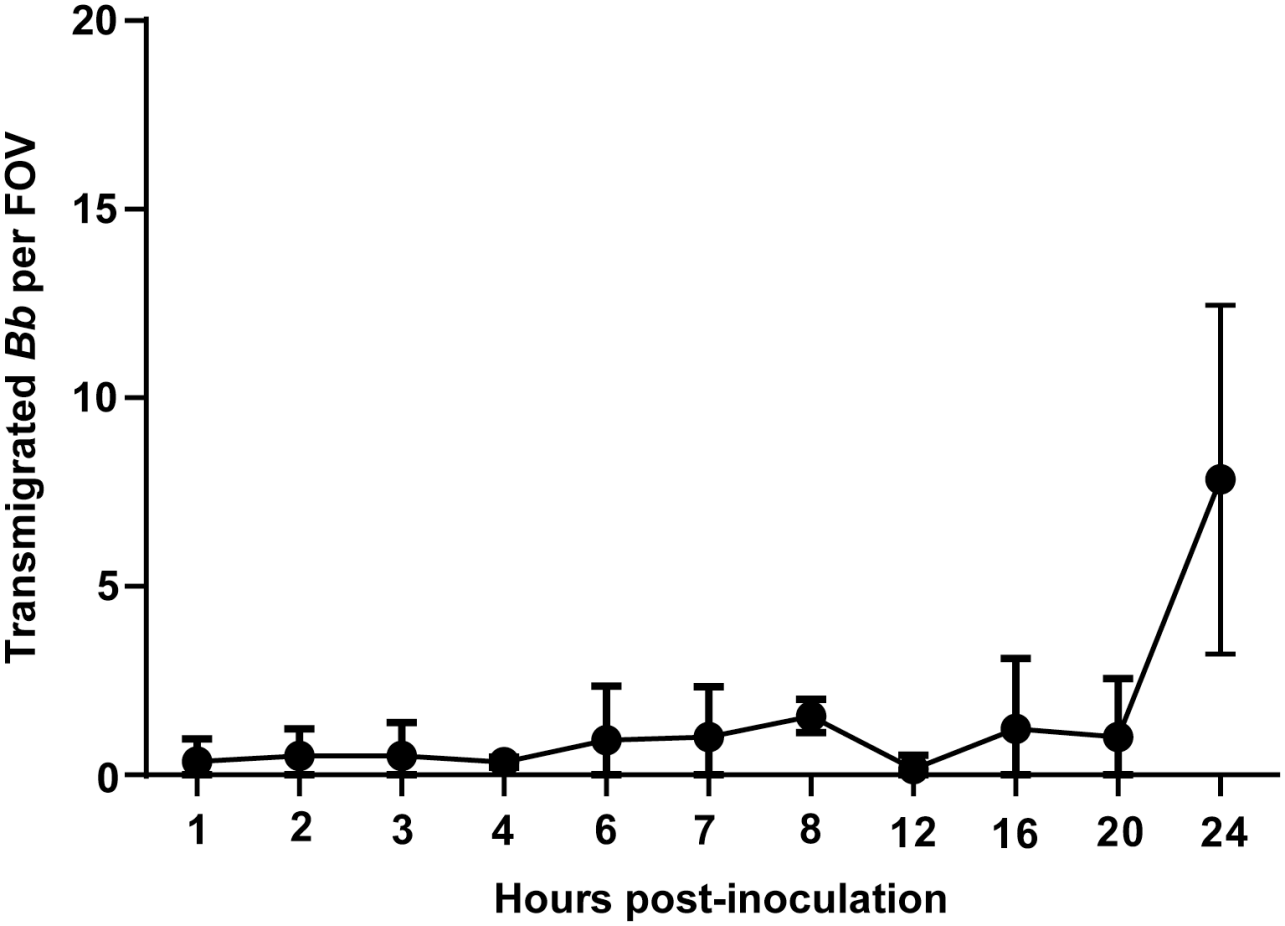
Kinetics of transmigration by fluorescent infectious *B*. *burgdorferi* in *Cd1d* ^*-/-*^ mice. Infectious GFP-expressing *B*. *burgdorferi* (GCB847) were injected into the tail vein of *Cd1d* ^*-/-*^ mice, 4x10^8^ spirochetes per mouse (n = 2/group for all time points except 20h where n = 6). At the indicated times vascular transmigration was scored in the knee joint-proximal tissue of living mice by intravital microscopy using a spinning disk laser confocal microscope. Blood vessels were stained with PE-conjugated PECAM-1 antibody. Green fluorescent spirochetes outside of the vasculature were counted in at least five fields of view (FOV) per mouse.

Mice were inoculated intravenously with 4 x 10^8^ spirochetes through the tail vein, and at 24 hours post-inoculation intravital microscopy was performed (see Materials and Methods) on the right knee of each infected mouse. As shown in Fig 2, intravital microscopy of GFP-expressing *B*. *burgdorferi* by spinning disk laser confocal microscopy revealed spirochetes that had escaped from the peripheral joint vasculature, which was labeled with a fluorescent anti-PECAM-1 antibody. These spirochetes displayed a to-and-fro translational motility (S1 and S2 Videos) and remained within the knee joint-proximal tissue, possibly as the result of a chemotactic response [25] to localized chemical stimuli. A kinetic analysis of spirochete transmigration (Fig 3) revealed that although low levels of transmigrated spirochetes were visible at a variety of time points, the highest observed levels were found at 24 hours post-inoculation, the necessary endpoint of these experiments due to constraints imposed on our animal protocol. This time point was used for subsequent analysis of spirochete transmigration.

### Analysis of peripheral knee vasculature transmigration levels of a *Δbbk32* mutant of *B*. *burgdorferi*

**Fig 4:**
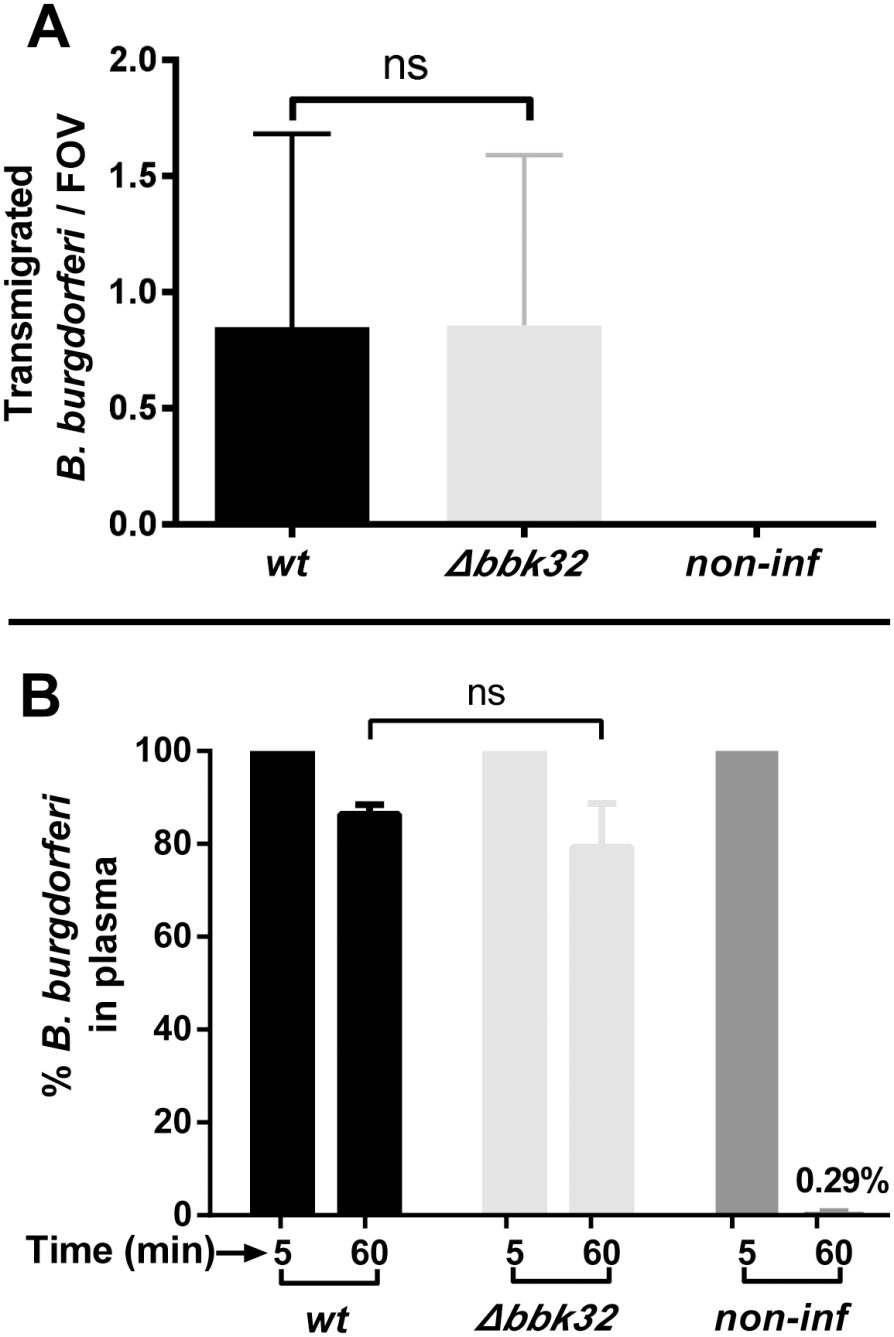
The effect of *bbK32* deletion in an infectious strain background on vascular transmigration and clearance in *Cd1d* ^*-/-*^ mice. **A)** GFP-expressing *B*. *burgdorferi* strains, infectious (GCB966), a *bbk32* deletion strain (GCB971) and a high-passage non-infectious strain (GCB706) were injected into the tail vein of *Cd1d* ^*-/-*^ mice (n = 6/group, 4x10^8^ spirochetes were injected per mouse). After 24 hours, vascular transmigration was scored in the knee joint-proximal tissue in the living mice by intravital microscopy using a spinning disk laser confocal microscope. Blood vessels were stained with PE-conjugated or Alexa Fluor 555-conjugated PECAM-1 antibody and green fluorescent spirochetes outside of the vasculature were counted in at least five fields of view (FOV) per mouse. Statistical significance was analyzed using the non-parametric Mann-Whitney test; ns denotes not significant (P-values >0.05). **B)** Concentrations of *B*. *burgdorferi* in mouse plasma after iv inoculation. *Cd1d* ^*-/-*^ mice were injected with *B*. *burgdorferi* through the tail vein and blood was withdrawn at 5 and 60 minutes post-inoculation (n = 3/group). Blood cells were allowed to settle overnight as described in Materials and Methods and spirochetes in the plasma were directly counted by dark-field microscopy. The change in spirochete concentration between 5 and 60 minutes was determined for each mouse as the percentage of spirochetes present at 60 minutes relative to the initial 5 minute time point. Statistical significance was analyzed using the non-parametric Mann-Whitney test; ns denotes not significant (P-values >0.05).

BBK32 is a *B*. *burgdorferi* adhesion that binds to fibronectin (Fn) and glycosaminoglycans (GAGs) [7, 19–21]. BBK32 promotes vascular tethering and dragging interactions in the vasculature of living mice [16] through endothelial interactions mediated by its Fn and GAG binding domains, respectively [15]. To determine if BBK32 plays an essential role in *B*. *burgdorferi* transmigration into joint-proximal tissue, we infected *Cd1d* ^*-/-*^ mice with wild-type and *Δbbk32* spirochetes expressing GFP (see S1 Table for strain information). Intravital microscopy was performed at 24 hours post-inoculation. The host vasculature was labeled with fluorescent antibody to PECAM-1 and video footage was collected on at least five areas in each knee during the one hour intravital experiment. Spirochetes that had transmigrated into the joint-proximal tissue were counted. As shown in Fig 4A, no difference was observed in the transmigration of *bbk32* versus wild-type spirochetes. In contrast, no detectable transmigration was observed using a fluorescent, non-infectious high-passage strain.

In our previous intravital studies on vascular adhesion, we noted that different *B*. *burgdorferi* strains and mutants sometimes displayed widely variable clearance rates from the vasculature [15]. Spirochete concentrations in the blood are certainly expected to influence early vascular interaction levels. However, their effect upon transmigration is currently unknown because transmigration is believed to be preceded by early stationary adhesion to the endothelium [14]. At 24 hours post-inoculation the number of circulating spirochetes has decreased by three orders of magnitude and is difficult to accurately determine. Moreover, with the low numbers of spirochetes in circulation at 24 hours, it is not possible to quantify the level of stationary adhesions. Nonetheless, large changes in spirochete clearance in mutant strains can be accurately assessed at one hour by direct microscopic counting. This was done by comparing blood samples obtained at five and 60 minutes post-inoculation. Sixty minutes was chosen as a general time point as the number of spirochetes was usually high enough for accurate counting and it allowed discrimination between very rapidly cleared and slowly cleared strains. Direct counting of spirochetes in plasma (see Materials and Methods) was performed by dark-field microscopy. Spirochete counts for each individual mouse at five minutes post-inoculation were defined as 100% and the percent spirochetes remaining for each mouse at 60 minutes was calculated. Fig 4B shows that the disruption of *bbk32* did not result in a change in the clearance rate of the mutant versus the wild-type, with approximately 80% of the spirochetes remaining in the circulation at one hour. In contrast, the high-passage non-infectious strain GCB706 was rapidly cleared. It is currently unknown if an increased clearance rate would affect transmigration levels, however, the absence of a change in the clearance rate of the *bbk32* mutant versus wild-type spirochetes eliminates the potential influence of circulating spirochete numbers from consideration as a factor in the influence of BBK32 on transmigration.

### Analysis of peripheral knee vasculature transmigration levels of a *Δp66* mutant and in *p66* mutants specifically deficient in integrin binding, in an infectious background

**Fig 5:**
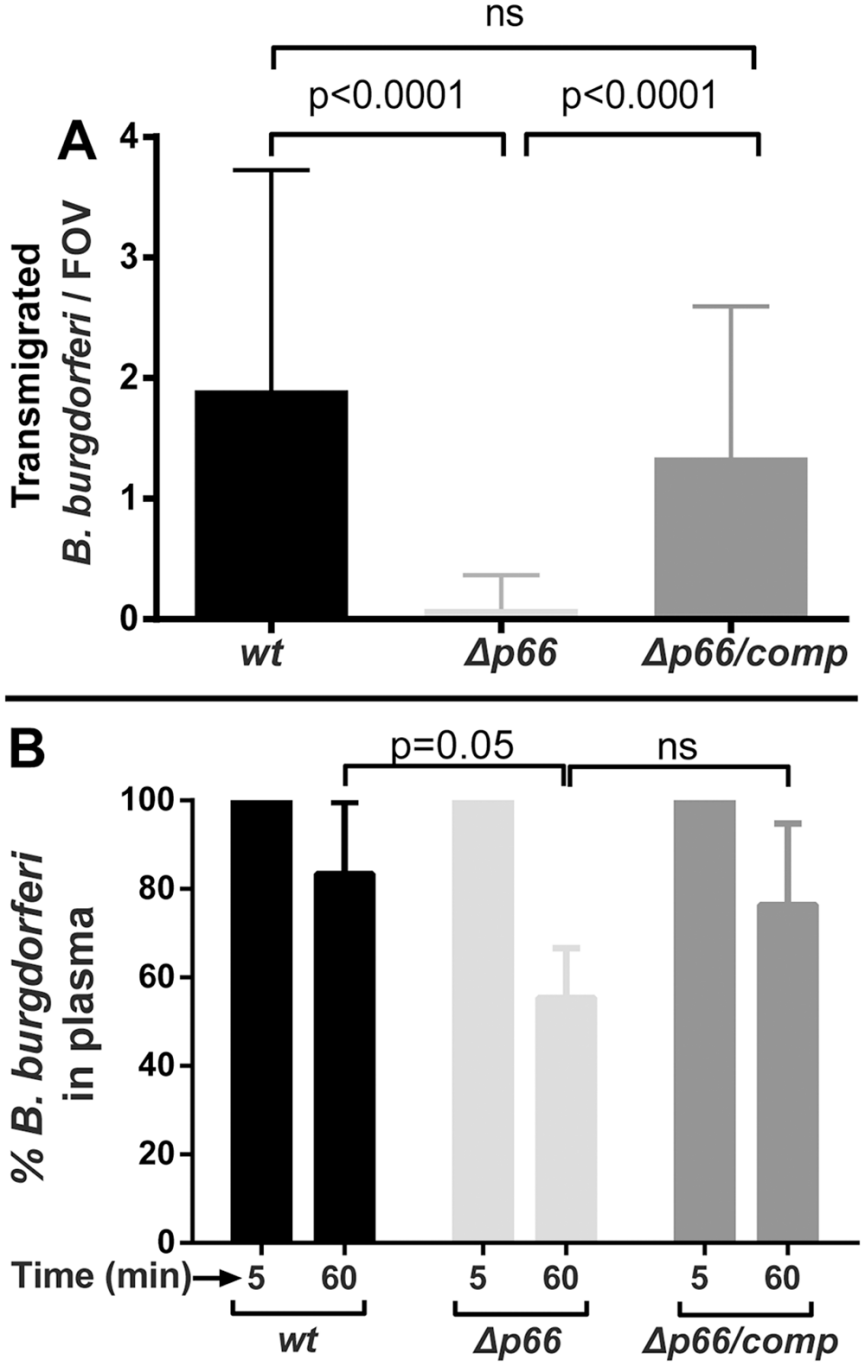
The effect of *p66* deletion in an infectious background on vascular transmigration and clearance in *CD1d* ^*-/-*^ mice. **A)** GFP-expressing *B*. *burgdorferi* strains, infectious wild type (GCB847), a *p66* deletion strain (GCB849) and a strain where the wild-type *p66* gene was reintroduced into the *p66* mutant (GCB851), were injected into the tail vein of *Cd1d* ^*-/-*^ mice (n = 6-16/group, 4x10^8^ spirochetes were injected per mouse). After 24 hours, vascular transmigration was scored in the knee joint-proximal tissue in the living mice by intravital microscopy using a spinning disk laser confocal microscope. Blood vessels were stained with PE-conjugated PECAM-1 antibody and green fluorescent spirochetes outside of the vasculature were counted in at least five fields of view (FOV) per mouse. Statistical significance was analyzed using non-parametric Kruskal-Wallis ANOVA followed by Dunn's multiple comparisons test. *P*-values for select pairwise comparisons are shown; ns denotes not significant (P-values >0.05). **B)**. Concentrations of *B*. *burgdorferi* in mouse plasma after iv inoculation. *Cd1d* ^*-/-*^ mice were injected with 4x10^8^ *B*. *burgdorferi* through the tail vein and blood was withdrawn at 5 and 60 minutes post-inoculation (n = 5-11/group). Blood cells were allowed to settle overnight as described in Materials and Methods and spirochetes in the plasma were directly counted by dark-field microscopy. The change in spirochete concentration between 5 and 60 minutes was determined for each mouse as the percentage of spirochetes present at 60 minutes relative to the initial 5 minute time point. Statistical significance was analyzed using non-parametric Kruskal-Wallis ANOVA followed by Dunn's multiple comparisons test. *P*-values for select pairwise comparisons are shown; ns denotes not significant (P-values >0.05).

P66 is a *B*. *burgdorferi* integrin adhesin [26–28] and porin [29–32] that is required for mouse infectivity [24]. The underlying reason for the lack of infectivity displayed by *B*. *burgdorferi* carrying a *p66* gene disruption is unknown. To determine if P66 plays an essential role in *B*. *burgdorferi* transmigration into joint-proximal tissue in the knee, we inoculated *Cd1d* ^*-/-*^ mice with wild-type infectious and *Δp66* spirochetes expressing GFP. Intravital microscopy was performed at 24 hours post-inoculation. As shown in Fig 5A, a dramatic and highly significant decrease in transmigration into the knee joint-proximal tissue was observed. This effect was specific to the *p66* mutation as demonstrated by restoration of transmigration by replacement of the disrupted *p66* gene with the wild-type allele. Analysis of the rate of clearance of the *p66* deletion strain revealed a clearance rate about twice as fast as the wild-type strain, with restoration of the wild-type clearance rate in the complemented strain. The mechanism of the more rapid clearance of the *Δp66* mutant is unknown and the effect of a clearance rate doubling on the formation of stationary adhesions leading to transmigration is unknown, but not expected to be responsible for the dramatic decrease in transmigration observed. However, it does raise a possible concern for the strength of the conclusion that can be drawn from this experiment.

**Fig 6:**
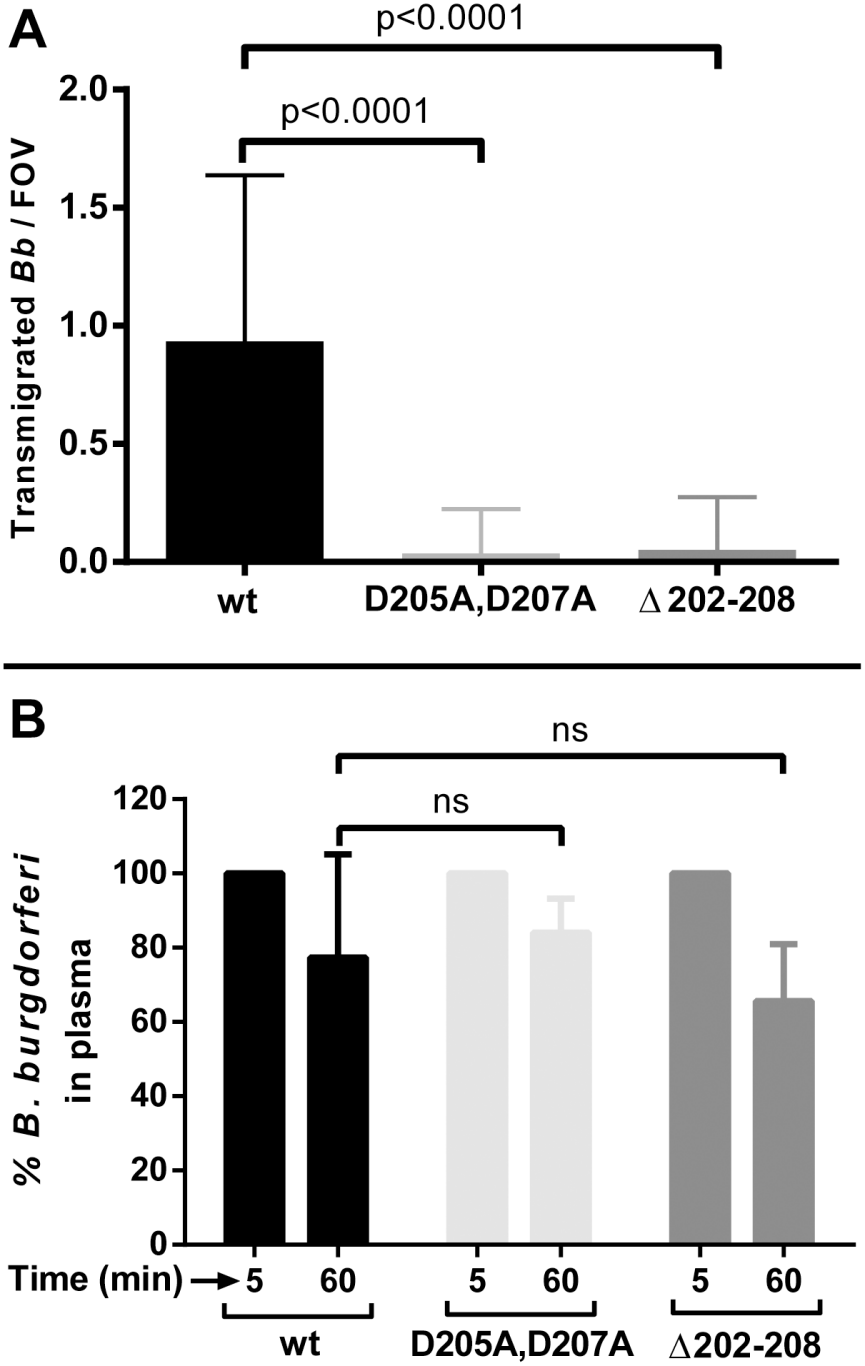
The effect of *p66* site-directed integrin binding mutants on vascular transmigration and clearance in *Cd1d* ^*-/-*^ mice. **A)** GFP-expressing *B*. *burgdorferi* strains, infectious wild type (GCB847), *p66* ^*D205A*,*D207A*^ (GCB3003) and *p66* ^*Δ202–208*^ (GCB3004) were injected into the tail vein of *Cd1d* ^*-/-*^ mice (n = 3/group, 4x10^8^ spirochetes were injected per mouse). After 24 hours, vascular transmigration was scored in the knee joint-proximal tissue in the living mice by intravital microscopy using a spinning disk laser confocal microscope. Blood vessels were stained with PE-conjugated PECAM-1 antibody and green fluorescent spirochetes outside of the vasculature were counted in at least five fields of view (FOV) per mouse. Statistical significance was analyzed using non-parametric Kruskal-Wallis ANOVA followed by Dunn's multiple comparisons test. *P*-values for select pairwise comparisons are shown; ns denotes not significant (P-values >0.05). **B)**. Concentrations of *B*. *burgdorferi* in mouse plasma after iv inoculation. *Cd1d* ^*-/-*^ mice were injected with *B*. *burgdorferi* through the tail vein and blood was withdrawn at 5 and 60 minutes post-inoculation (n = 3/group). Blood cells were allowed to settle overnight as described in Materials and Methods and spirochetes in the plasma were directly counted by dark-field microscopy. The change in spirochete concentration between 5 and 60 minutes was determined for each mouse as the percentage of spirochetes present at 60 minutes relative to the initial 5 minute time point. Statistical significance was analyzed using non-parametric Kruskal-Wallis ANOVA followed by Dunn's multiple comparisons test. *P*-values for select pairwise comparisons are shown; ns denotes not significant (P-values >0.05).

To further investigate the mechanism underlying the decreased transmigration of the *p66* mutant, we assessed the transmigration of two site-directed mutants, *p66* ^*D205A*,*D207A*^ and *p66* ^*Δ202–208*^ [33]. The proteins encoded by these genes are either full length or have a seven amino acid deletion, respectively, in contrast to the *Δp66 strain*, which has no P66 on the cell surface. Both mutant proteins are specifically defective in integrin-binding activity, but are localized on the surface of *B*. *burgdorferi* and retain channel-forming activity [33]. As shown in Fig 6, both P66 integrin binding mutants displayed a dramatic and highly significant reduction in transmigration into joint-proximal tissue by the intravital microscopic assay 24 hours after inoculation of *Cd1d* ^*-/-*^ mice. Moreover, in contrast to the P66 full deletion mutant, both site-directed mutants displayed clearance rates from the vasculature that did not differ significantly from that of wild-type *B*. *burgdorferi*. The two site-directed P66 mutants, therefore, provide more convincing data for an essential role for P66, and in particular the integrin-binding residues, in the transmigration process.

**Fig 7:**
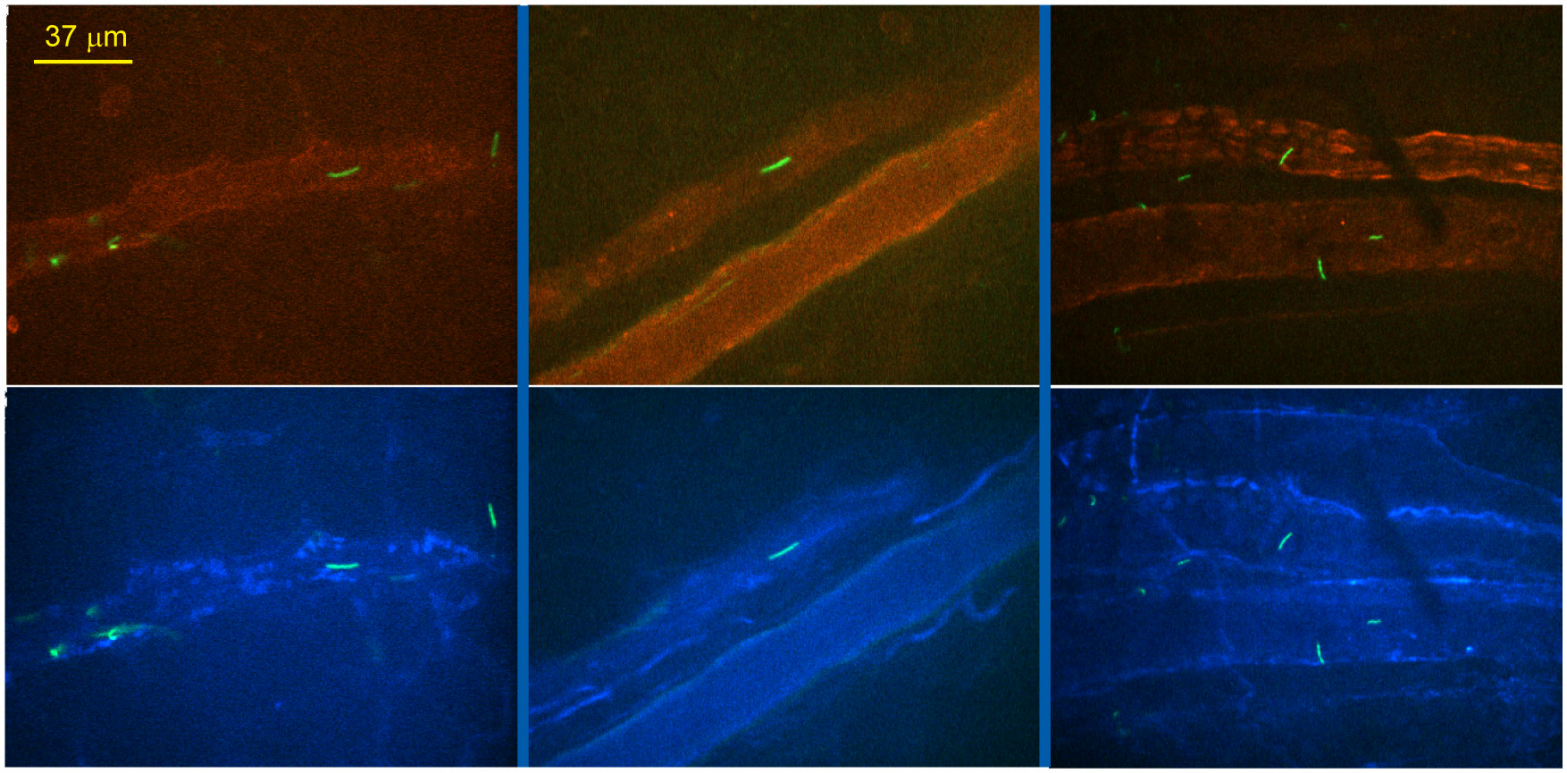
Visualization of β_3_ integrin in post-capillary venules in knee joint-proximal tissue by multi-channel spinning disk intravital microscopy. GFP-expressing infectious *B*. *burgdorferi* (GCB847) was injected into the jugular vein of BALB/c mice as noted for other intravital experiments and data were acquired between 5–60 minutes postinfection. Blood vessels were stained with PE-conjugated PECAM-1 antibody and Alexa Fluor 647-conjugated β_3_ integrin antibody. The upper panel shows tethering and stationary interactions of *B*. *burgdorferi* (green), with PECAM-1 expressing endothelium visualized inside the blood vessels (red). The lower panel shows tethering and stationary interactions of the *B*. *burgdorferi* (green), with β_3_ integrin visualized inside the blood vessels (blue). The β_3_ integrin staining is also visible in smooth muscles (blue) surrounding the blood vessels.

The corollary of the conclusions from Fig 6 is that integrins bound by P66 are expressed on the surface of the endothelium in post-capillary venules. To explore this, we visualized β_3_ integrins on the surface of endothelial cells by multi-channel spinning disk intravital microscopy. Fig 7 shows micrographs with spirochetes and PECAM-1 (green and red, respectively) visualized in the top panels. The bottom panels show the same micrographs with spirochetes and β_3_ integrins (green and blue, respectively) visualized. In general, a uniform pattern of staining with the anti-integrin antibody is seen, along with areas of more pronounced staining, similar to that observed for PECAM-1. However, specific areas of integrin concentration differ from those observed with PECAM-1. Spirochete binding did not occur in areas of integrin concentration, but was found in areas with uniform integrin expression.

From the data in Figs 5, 6 and 7 we conclude that P66 is required for efficient transmigration into the joint-proximal tissue of the knee and that the integrin-binding activity of the protein plays an integral role in this process.

### Analysis of vascular adhesion of a non-infectious *Δp66* strain

**Fig 8:**
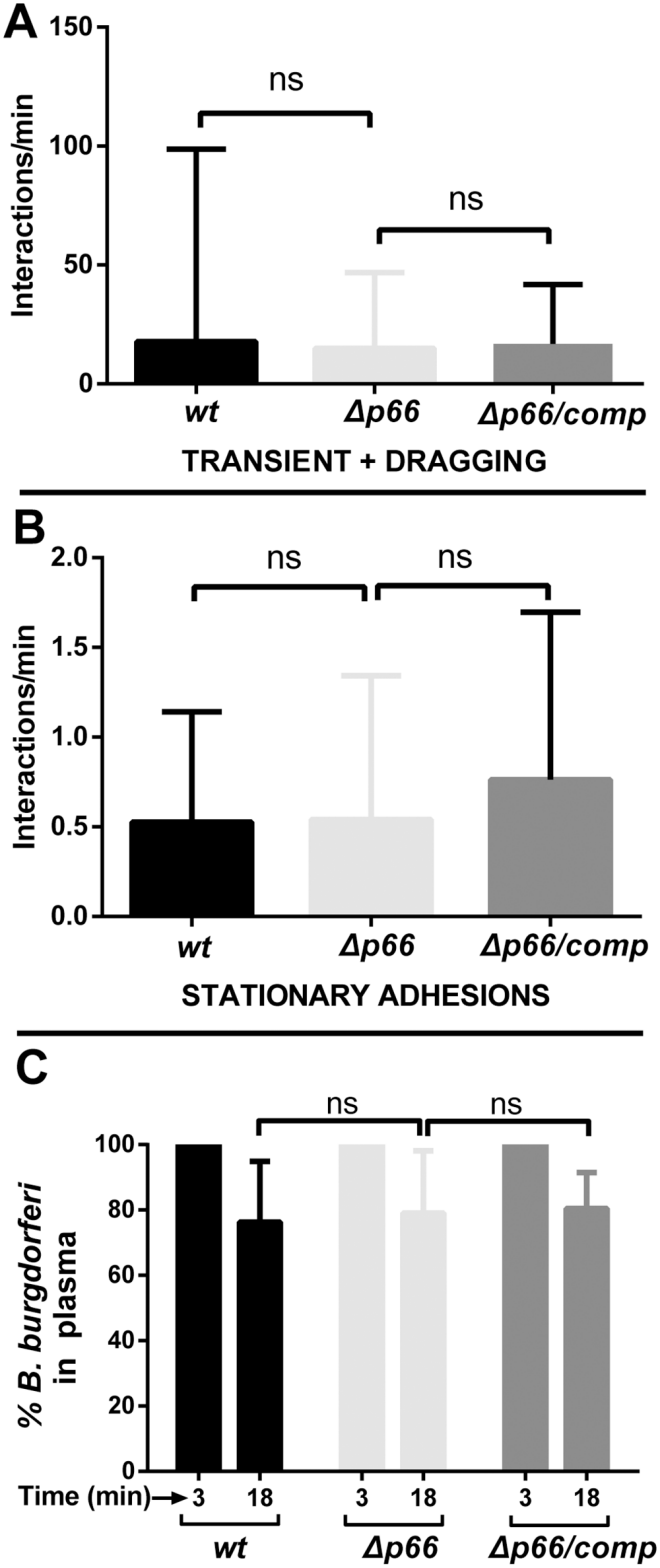
The effect of *p66* deletion on vascular adhesion and clearance in a high- passage *B*. *burgdorferi* strain in BALB/c mice. Non-infectious GFP-expressing *B*. *burgdorferi* wild type (GCB3212), *Δp66* (GCB3214), and a strain where the wild-type *p66* gene was reintroduced into the *p66* deletion mutant *(Δp66/comp*, GCB3218) were injected into the jugular vein of BALB/c, 4x10^8^ spirochetes per mouse (n = 7/group). Over a period of up to 45 minutes, microvascular interaction rates **A)** (tethering + dragging), and stationary adhesions **B)** were enumerated in the knee joint-proximal tissue by intravital microscopy using spinning disk laser confocal microscopy as described in Materials and Methods. Blood vessels were stained with PE-conjugated PECAM-1 antibody. Statistical significance was analyzed using the non-parametric Kruskal-Wallis test; ns denotes not significant (P-values >0.05). **C)** Concentrations of *B*. *burgdorferi* in mouse plasma after iv inoculation. BALB/c mice were inoculated with *B*. *burgdorferi* through the tail vein as above and blood was withdrawn at 3 and 18 minutes post-inoculation (n = 7/group). Blood cells were allowed to settle overnight as described in Materials and Methods and spirochetes in the plasma were directly counted by dark-field microscopy. The change in spirochete concentration between 3 and 18 minutes was determined for each mouse as the percentage of spirochetes present at 18 minutes relative to the initial 3 minute time point. Statistical significance was analyzed using the non-parametric Kruskal-Wallis test; ns denotes not significant (P-values >0.05).

Because of the requirement for P66 and its integrin binding sequences for transmigration in peripheral knee vasculature, it was of interest to determine whether P66 can promote vascular adhesion, an early stage of the transmigration process that can be monitored by intravital microscopy in a living mouse [14–16]. A complicating factor in designing such an experiment was that early *B*. *burgdorferi-*microvasculature interactions are mediated predominantly by BBK32 and one or more unknown fibronectin-independent adhesins [14, 15]. In the presence of these adhesins, the effect of other adhesins with less dramatic endothelial-binding properties is obscured. We, therefore, introduced the *Δp66* mutation into the non-infectious strain GCB706, which is a fluorescent version of the high passage strain B31-A (see S1 Table) that lacks expression of *bbk32* [15, 16]. This wild-type non-infectious strain background, therefore, displays only low levels of spirochete-endothelial interactions in post-capillary venules. For this experiment, the spirochetes were grown without blood supplementation to avoid induction of other possible adhesins. As shown in Fig 8, intravital microscopy was used to monitor tethering plus dragging (Fig 8A) and stationary attachment (Fig 8B) of the wild-type parent, the *Δp66* mutant and the complemented mutant in the peripheral knee vasculature. No difference in microvascular interactions (tethering and dragging interactions combined) was observed between wild type and the *Δp66* mutant or the *Δp66* mutant and the *Δp66/*complemented strain (Fig 8A). Similarly, there was no difference in stationary adhesions between the wild-type and the mutant strain or the mutant strain and the complemented mutant (Fig 8B). Finally, the vascular clearance rates of all three strains were the same (Fig 8C). These results indicate that P66 does not promote the early transient or stationary interactions that withstand the shear force of vascular flow. Instead, *B*. *burgdorferi* relies upon other adhesins to promote initial microvascular-endothelial interactions, which facilitates subsequent P66-integrin contacts leading to transmigration.

## Discussion

In this work we report a new approach to study vascular transmigration. This approach utilizes assessment of transmigration in the mouse peripheral knee vasculature by intravital microscopy in *Cd1d* ^*-/-*^ mice. The intravital methodology allows direct visualization and enumeration of transmigrated spirochetes in living mice. The use of *Cd1d* ^*-/-*^ mice makes this assay possible by eliminating iNKT cells, a component of the innate immune response that disrupts dissemination of *B*. *burgdorferi* into mouse joints through a granzyme dependent pathway [17, 18]. The peripheral knee vasculature also provides a desirable target for observation since transmigrating spirochetes remain in the local environment, in contrast to flank skin where they rapidly leave the transmigration site [14]. The general applicability to other tissues of conclusions drawn herein from intravital data obtained from the peripheral knee vasculature is currently unknown as this work is the first reported study of spirochete transmigration using intravital microscopy and the peripheral knee vasculature currently the only location where quantitative imaging of this process is currently viable.

Using intravital microscopy of *Cd1d* ^*-/-*^ mice inoculated with GFP-expressing *B*. *burgdorferi*, we have monitored the effect of two important spirochete adhesins on transmigration into the joint-proximal tissue of the mouse knee. The Fn and GAG binding protein, BBK32, is believed to be involved in early steps of the dissemination process. It is sufficient to restore microvascular interactions to a non-adherent, non-infectious, high-passage *B*. *burgdorferi* strain [16]. BBK32 mediates tethering and dragging interactions with the host microvasculature through its Fn and GAG binding domains, respectively [15]. These interactions are believed to precede transmigration. Interestingly, disruption of the gene for BBK32 did not result in a decrease in transmigration into the mouse knee joint-proximal tissue and the mutant spirochetes were not cleared significantly faster than the wild-type parent. The absence of a transmigration phenotype for the *bbk32* mutant is likely the result of duplicity of function; in the absence of BBK32, an unknown Fn-independent adhesin (or adhesins) maintain microvascular interactions in the mouse peripheral knee vasculature at 50% of the wild-type level [15]. We predict that the combined disruption of both BBK32 and the unknown Fn-independent adhesin(s) would dramatically impact transmigration; however, such an experiment is precluded until the identity of the unknown Fn-independent adhesin(s) is established.

It is also noteworthy that in contrast to a lack of an effect of a *bbk32* mutation on transmigration by our intravital assay, it has been previously reported that a *bbk32* mutation results in a decreased ability to invade joints as determined by bioluminescent imaging, qPCR and spirochete recovery by culture [10]. An explanation for the discrepancy with our results is that the previous study utilized intradermal needle inoculation of mice with 10^3^−10^5^ spirochetes and followed outcomes up to 14 days post-infection. Although not natural tick inoculation, this mouse model has been used for decades to study *Borrelia* infections. In contrast, to obtain mechanistic data on the transmigration process, we have used the reductionist approach of intravenous inoculation of 4x10^8^ spirochetes in Cd1d^-/-^ mice for intravital imaging of transmigration at 24 hours. Our approach seeks to identify molecules directly involved in the transmigration process and seeks to eliminate other possible roles of BBK32 in the early infection process. The variance between our results and those previously published [10] suggests that BBK32 may have other functions in the infection process in wild-type mice that our assay has sidestepped, but which are noticeable in the traditional animal model. Alternatively, the variance may result from differences in gene expression associated with host adaptation of the spirochetes within the timeframes of the two experiment [34–38].

In contrast to the BBK32 result, disruption of the gene encoding the integrin adhesin and porin, P66, resulted in a dramatic reduction in transmigration. Moreover, mutants with either alanine substitutions for the aspartic acids at position 205 and 207 or deletion of residues 202–208, also resulted in a dramatic loss of transmigration ability. The noted mutations specifically target the integrin-binding residues of P66 without affecting its porin activity [33]. Our data therefore suggest that integrin binding is an essential feature of *B*. *burgdorferi* transmigration. Analysis of the effect of *p66* disruption on vascular adhesion in a low-adherence high passage strain (lacking BBK32) revealed that P66 does not promote early transient interactions or stationary adhesions. Therefore, the transmigration process appears to be temporally choreographed with adhesins such as BBK32 and the unknown fibronectin-independent adhesion(s) playing an initial role of establishing *B*. *burgdorferi-*microvascular interactions that subsequently facilitate P66-integrin communication leading to transmigration through an as yet undefined process. In agreement with the findings presented here are the recent results that the *p66* ^*Δ202–208*^ mutant displays undetectable bacterial burdens at two weeks after iv inoculation and reduced migration across microvascular endothelial monolayers *in vitro* [33].

A variety of bacterial pathogens encode integrin-binding proteins (see [39, 40]). Integrin binding plays diverse roles for bacterial pathogens, including as an adherence target on host cells and extracellular matrix (ECM). Integrin binding also functions to promote pathogen-host signaling pathways that may lead to cellular invasion [39, 40]. However, a function for an integrin-binding protein in bacterial vascular transmigration has not been previously reported. The slowly emerging picture of *B*. *burgdorferi* transmigration is that it is a multi-step process involving several *B*. *burgdorferi* proteins and host molecules [14–16] and has general similarities to leukocyte transmigration [41–43], although very significant deviations exist. In both cases the process is initiated by tethering interactions that withstand the shear stress of vascular flow. In *B*. *burgdorferi* this occurs between the Fn-binding domain of BBK32 and endothelial surface localized Fn. Subsequently, rolling (leukocytes) or dragging (*B*. *burgdorferi*) occurs. In Lyme disease spirochetes the dragging stage involves additional interactions between the GAG-binding domain of BBK32 and glycosylated endothelial receptors. Thereafter, stationary adhesion occurs, which involves at least one additional spirochete factor [14–16]. Our current work suggests that P66 is involved upon the establishment of a stationary interaction, where P66-integrin interactions can occur within an established *B*. *burgdorferi*-endothelial complex.

Tethering, dragging and stationary *B*. *burgdorferi* interactions can be observed within minutes of iv spirochete injection. However, transmigration occurs only at background levels until about 24 hours after iv injection. The velocity of *B*. *burgdorferi* in liquid or 2–3% gelatin is about 3.5–5 μm per second [44, 45]. The rate of forward motion of spirochetes escaping postcapillary venules is much slower, about 3.4 μm per minute. Even at this slower velocity the average time span of spirochetal escape is about 11 minutes [14]. This raises the question of why only background levels of transmigration occur until 24 hours post-inoculation when both adhesion and physical escape can occur within a much shorter time-frame. A possible explanation for this that there may be a requirement for stationary *B*. *burgdorferi* adhesions to promote localized endothelial activation resulting in an increased permeability of the endothelium that facilitates spirochete transmigration. Conversely, stable adherence of *B*. *burgdorferi* to the endothelium may promote activation of the spirochete by inducing the production of factors that are necessary for transmigration. In the case of leukocytes, activation of integrins precedes an increase in ligand affinity, resulting in stationary adhesion and migratory arrest on the endothelial surface, although integrins also play other roles in leukocyte transmigration [41–43]. In contrast, *B*. *burgdorferi* does not contain integrins, but encodes P66, an integrin adhesin and porin. We show here that the integrin-binding region of P66 is required for transmigration into the mouse knee joint-proximal tissue. Whether the porin activity is also required for transmigration remains unknown at present since P66 mutants affecting the porin but not the integrin-binding activity do not currently exist. The mechanism by which P66 is involved in the transmigration process awaits further characterization. Finally, it is important to note that the intravital imaging studies reported here provide a powerful tool for studying the role of P66 in vascular interactions and transmigration, however, they do not shed light on other roles of P66 in the natural infectious process as recently reported [33]. The work reported here describes a new approach to study vascular transmigration of the Lyme disease spirochete, which has resulted in the identification of the first *B*. *burgdorferi* adhesin believed to be directly involved in vascular transmigration.

## Materials and Methods

### Ethics statement and use of mice

All animal experimentation was carried out in accordance with the principles outlined in the most recent policies and *Guide to the Care and Use of Experimental Animals* by the Canadian Council on Animal Care. The animal protocol (AC12-0218) was approved by The Animal Care Committee of the University of Calgary. Wild-type BALB/c mice were purchased from Charles River (Wilmington, MA) and *CD1d* ^*-/-*^ mice in a BALB/c background (Jax #2962) were bred in-house at the Clara Christie Centre for Mouse Genomics at the University of Calgary. Mice of both genders between 5–16 weeks of age were used.

### Bacterial strains and culture

The GFP-expressing *B*. *burgdorferi* strains used in this study are described in S1 Table. pTM61-*strep* (Sm^R^/Sp^R^) was constructed by digesting pTM61 [14] with AvrII and MluI (New England Biolabs, Beverly, MA), then dephosphorylated using alkaline phosphatase. The spectinomycin/streptomycin resistance cassette [46] was amplified from pTAspc (provided by Dr. D. Scott Samuels) using the primers flgBpAvrII (CCTAGGTAATACCCGAGCTTCAAGGAAG) and aadAMluI (ACGCGTGACGTCATTATTTGCCGACTACC). The PCR product was cloned directly in pGEM T Easy (Promega, Madison, WI), then excised with AvrII and MluI and ligated into the pTM61 prepared as described above. The ligation mixes were used to transform *E*. *coli* strain Top10 (Life Technologies, Grand Island, NY) by electroporation [47]. Selection of the desired recombinant plasmid was performed by plating on Luria broth agar supplemented with spectinomycin at 80 μg/ml. For further experiments in *B*. *burgdorferi*, the plasmid was selected for using 80 μg/ml of streptomycin in Barbour-Stoenner-Kelly II (BSK-II) medium (with 6% rabbit serum, Cedarlane Laboratories ltd., Burlington, ON). Plasmid content was analyzed for all constructed strains by multiplex PCR [48].

Spirochete cultures in BSK-II medium were inoculated from frozen glycerol stocks. Levels of antibiotics used are indicated in S1 Table. The spirochete cultures were grown at 35°C to a concentration between 1–5 x 10^7^/ ml. Spirochetes were then diluted to 1 x 10^5^/ml in 100 ml BSK-II medium and grown for 24 hours, followed by 48 hours growth with 1% BALB/c mouse blood (vol/vol) and 1x *Borrelia* antibiotic mixture (final concentrations of 20 μg/ml phosphomycin, 50 μg/ml rifampicin, and 2.5 μg/ml amphotericin B). The bacterial densities were not allowed to exceed 5 x 10^7^/ml. Spirochetes were harvested by centrifugation at 6000xg for 15 minutes at 4°C and washed twice with 100 ml of cold phosphate-buffered saline (PBS).

### Mouse infection

BALB/c or *CD1d* ^*-/-*^ mice were anesthetized by intraperitoneal injection of 200 mg/kg ketamine hydrochloride (Bimeda-MTC, Animal Health Inc., Cambridge, ON) and 10 mg/kg of xylazine hydrochloride (Bayer Inc., Toronto, ON). For vascular transmigration assays, anesthetized *CD1d* ^*-/-*^ mice were secured in a mouse restrainer and inoculated by tail vein injection with 4 x 10^8^ spirochetes in 150 μl of phosphate buffered saline (PBS). For vascular adhesion assays, anesthetized mice were intravenously injected with 4 x 10^8^ spirochetes via a jugular vein catheter.

### Direct counts of spirochetes in plasma

Mice were anesthetized as described above. Additional intraperitoneal injection of anesthetic was done as necessary to maintain anesthesia for the total duration of the procedure. For direct spirochete counts in the vascular transmigration assays, blood samples (35 μl, collected in an equal volume of 20mM sodium citrate) were taken from the saphenous or tail vein at five and 60 minutes post-inoculation. For counts in vascular adhesion assays, samples were collected at three and 18 minutes post-inoculation. Samples were drawn into glass capillary tubes, the bottom of the capillary tubes were blocked with vacuum grease and incubated vertically at 4°C overnight to allow separation of blood cells by gravity. The next day, the glass capillaries were cut with a diamond pencil near the white blood cell-plasma interphase and 25 μl of plasma was collected and centrifuged at 6000xg for 15 minutes at 4°C to pellet the spirochetes. The supernatant fluid was carefully decanted and discarded. The pellet was resuspended in 6 μl of PBS, diluted as necessary, and loaded on a Petroff-Hausser counting chamber and counted. Spirochete numbers for each mouse at a later time point (18 or 60 minutes post-inoculation) were expressed as a percentage of the spirochete numbers observed at an initial time point (3 or 5 minutes post-inoculation) for each individual mouse.

### Surgical preparation for jugular vein catheterization and knee intravital microscopy

Deep surgical anesthesia in BALB/c or *CD1d* ^*-/-*^ mice was induced as described above and maintained by regular iv injections of anesthetic in sterile normal saline. A home-made catheter made of PE10 polyethylene tubing (I.D. 0.011" O.D. 0.024", Intramedic, Becton Dickinson and Company, Sparks, MD) was used as a jugular vein catheter. The catheter was connected to a 1ml syringe filled with 100 I.U. / ml of heparin in physiological saline solution. After confirmation of induction of surgical anesthesia, the knee was prepared for intravital microscopy as previously described [49]. The hair over the jugular vein area was clipped and the mouse was positioned in dorsal recumbency, and secured over a regulated heating pad. The skin was incised at the ventral cervical region right to the midline at the level of the clavicle bone. Under a dissection microscope, the right jugular vein was separated carefully from adjacent fat and connective tissues by blunt dissection and the anterior aspect of the jugular vein was tied with 4.0 surgical sutures and secured tightly to the imaging platform with surgical tape. A slightly bent 30½ G needle was inserted parallel in the jugular vein and the vein was slightly lifted up gently with help of the inserted needle. A PE10 polyethylene tube was introduced into the vein with simultaneous removal of the needle from the vein. The jugular vein patency was confirmed by withdrawal of blood in the tubing and the catheter was secured at cranial and distal locations with a double surgical knot around the vein. The jugular vein was used for maintaining the anesthesia level, injecting fluorescent antibodies, and injecting spirochetes. For knee preparation, the right hind limb was slightly flexed and secured to a home-made plastic knee holder using a surgical tie just above the metatarsal joint. The medial aspect of the knee joint was slightly oriented upwards, placed, and immobilized in the plastic knee holder. The hair was clipped, the area was swabbed with mineral oil, and the skin over the medial aspect of the knee joint was carefully removed by incision. The connective tissues over the exposed area was carefully removed without injuring blood vessels. The exposed area was always immersed in isotonic saline to keep it moist. A glass cover slip was positioned over the exposed joint area and secured to the knee-holder using vacuum grease.

### Spinning disk confocal intravital microscopy

Spinning disk confocal microscopy was performed using an Olympus BX51W1 base (Olympus, Center Valley, PA) fitted with 10x/0.30 UPlanFLN air and 20x/0.95 XLUMPlanFI water immersion objectives. The microscope was equipped with a confocal light path (WaveFx, Quorum, Guelph, ON, Canada) based on a modified Yokogawa CSU-10 head (Yokogawa Electric Corporation, Tokyo, Japan). The mice were injected with antibodies immediately before i.v. inoculation with *B*. *burgdorferi*, which corresponded to roughly 10–20 minutes before imaging, depending upon the experiment. The endothelial cells were stained with phycoerythrin (PE)-conjugated anti-PECAM-1 (5 μg per mouse; clone MEC 13.1; BD Biosciences, Mississauga, ON) or PECAM-1 antibody (50 μg per mouse; clone MEC 13.3; BD Biosciences, Mississauga, ON) conjugated in-house to Alexa Fluor 555 (Invitrogen, Burlington, ON) or Alexa Fluor 594 anti-mouse CD31 (5 μg per mouse; clone MEC 13.3; BioLegend Inc, San Diego, CA). β_3_ integrin staining was achieved using Alexa Fluor 647 anti-mouse/rat CD61 antibody (7.5 μg per mouse; clone 2C9.G2; BioLegend Inc, San Diego, CA). The spirochetes were engineered for GFP expression as described earlier. Vascular transmigration was evaluated by exciting the fluorophores with two laser wavelengths (488 nm and 561 nm; Cobolt, Stockholm, Sweden) and visualizing with proper band-pass filters (Semrock, Rochester, NY). A back-thinned electron-multiplying charged-coupled device camera (512 x 512 pixels; C9100-13, Hamamatsu, Bridgewater, NJ, USA) was utilized for detecting emitted fluorescence. Volocity software (version 6.0.1, Improvision, Lexington, MA) was used to control the microscope, image acquisition, and analysis. The sensitivity settings for the red and green laser used was 255 with the autocontrast setting turned on. All laser power settings were set to the highest, i.e. 1.84 mv. For video acquisition, the shutter setting was selected as ‘do not manage shutters’ and binning settings were set to 2x for GFP and RFP channels, which allowed data acquisition at a rate of between 14–20 frames per second.

### Intravital vascular transmigration assay

The vascular transmigration assay for *B*. *burgdorferi* was done in deeply anesthetized *CD1d* ^*-/-*^ mice with the medial aspect of the knee surgically prepared for imaging as described above. For transmigration assays, the *CD1d* ^*-/-*^ mice were intravenously inoculated with spirochetes 1–24 hours before imaging as described above. Images were acquired using a 10x objective lens and spirochetes were counted manually in the field of view (FOV). The data were pooled from a total of 6–16 mice per group from two independent experiments. Five fields of view or more were counted for each mouse. Because of the variability of intravital results that can be observed in different experiments, all *B*. *burgdorferi* strains being compared were analyzed in the same intravital session (eg, in Fig 5 one mouse was analyzed for each *B*. *burgdorferi* strain in each intravital session).

### Intravital vascular adhesion assay

The vascular adhesion assay was done in deeply anesthetized *CD1d* ^*-/-*^ mice with the medial aspect of the knee surgically prepared for imaging as described above. For vascular adhesion assays, BALB/c mice were intravenously inoculated via the jugular vein catheter with spirochetes 10 minutes before imaging. Vascular interactions were recorded 18–45 minutes post-inoculation from straight, unbranched post-capillary venules of 15–25 μm diameter in the knee. Videos were recorded through 20x/0.95 XLUMPlanFI water immersion objectives. Spirochete-vascular interactions were counted in a length of 100 μm along the blood vessel manually as described before [14–16]. Because of the variability of intravital results that can be observed in different experiments, all *B*. *burgdorferi* strains being compared were analyzed in the same intravital session (eg., in Fig 5 one mouse was analyzed for each *B*. *burgdorferi* strain in each intravital session).

### Statistics

GraphPad Prism 5.0 (GraphPad Software Inc., San Diego, CA) was used for statistical data analysis. Error bars in all graphs denote the standard deviation. Statistical significance was computed for experiments as noted in each figure legend.

## Supporting Information

S1 Table *B*. *burgdorferi* strains used. (DOCX)

S1 Video Spinning disk confocal intravital microscopy footage of fluorescent *B*. *burgdorferi* transmigrated into knee joint-proximal tissue of *Cd1d* ^*-/-*^ mice. Experimental conditions are as per Figs 2–4. The video was captured 24 hours after tail vein injection of each mouse with 4x10^8^ GFP-expressing *B*. *burgdorferi* (GCB726). The vasculature was counterstained with Alexa Fluor 555-conjugated PECAM-1 antibody. Elapsed time is shown at the top right and the scale is at the bottom left.
(MOV)

S2 Video Spinning disk confocal intravital microscopy footage of fluorescent *B*. *burgdorferi* transmigrated into knee joint-proximal tissue of *Cd1d* ^*-/-*^ mice. Experimental conditions are as per Figs 2–4. The video was captured 24 hours after tail vein injection of each mouse with 4x10^8^ GFP-expressing *B*. *burgdorferi* (GCB726). The vasculature was counterstained with Alexa Fluor 555-conjugated PECAM-1 antibody. Elapsed time is shown at the top right and the scale is at the bottom left.
(MOV)
