# Supplementary material for: Intravital Imaging of Vascular Transmigration by the Lyme Spirochete: Requirement for the Integrin Binding Residues of the B. burgdorferi P66 Protein
Source: PLoS Pathog. 2015 Dec 18;11(12):e1005333. doi: 10.1371/journal.ppat.1005333 (PMC4686178; doi:10.1371/journal.ppat.1005333)
Supplement: S1 Table — (DOCX) [file ppat.1005333.s001.docx]

| **Strain number** | | **Background/**  **Reference** | **Description** | **Antibiotic resistance** | **Source/**  **Reference** |
| --- | --- | --- | --- | --- | --- |
| GCB706 | | B31-A  [1] | B31-A + pTM61 (GFP, Gm^R^) | Gent 100 µg/ml | [2] |
| GCB726 | | 5A4 NP1  [3] | 5A4 NP1 (Km^R^)+ pTM61 (GFP, Gm^R^), clone 2 | Gent 100 µg/ml | [4] |
| GCB847 | | B31 A3  [5] | B31 A3 + pTM61 (GFP, Gm^R^), clone 23 | Gent 100 µg/ml | This work |
| GCB849 | | B31 A3 | *Δp66::kan* (K04 C3-14) + pTM61-*strep* (GFP, Sm^R^), clone 1 | Kan 200 µg/ml Strep 50 µg/ml | [6], This work |
| GCB851 | | B31 A3 | *Δp66::kan* (K04 C3-14) +*p66* restored to chromosome (Gm^R^) C3-14^cc^ clone 23 + pTM61-*strep* (GFP, Sm^R^), clone 3 | Gent 100 µg/ml Strep 50 µg/ml | [7], This work |
| GCB966 | | B31 ML23  [8] | ML23 + pJW201 (GFP, Gm^R^) | Gent 100 µg/ml | [9] |
| GCB971 | | B31 ML23 | JS315/pJW201 *Δbbk32::strep ­*+ pJW201 (GFP, Gm^R^) | Strep 50 µg/ml Gent 100 µg/ml | [9] |
| GCB3003 | | B31 A3 | B31 A3 K04 C3-14 + *p66^D205A,D207A^* restored to chromosome clone 2-30 + pTM61-*strep* (GFP, Sm^R^), clone 2-1 | Gent 40 µg/ml  Strep 80 µg/ml | [7], This work |
| GCB3004 | B31 A3 | B31 A3 K04 C3-14 + p66^Δ202-208^ restored to chromosome clone 29 + pTM61-*strep* (GFP, Sm^R^), clone 3-1 | Gent 40 µg/ml  Strep 80 µg/ml | [7], This work |  |
| GCB3212 | B31-A | B31-A + pTM61-*strep* (GFP, Sm^R^) | Strep 50 µg/ml | This work |  |
| GCB3214 | B31-A | *Δp66::kan* (K04) into GCB3212 +pTM61-*strep* (GFP, Sm^R^) | Kan 200 µg/ml  Strep 50 µg/ml | This work |  |
| GCB3218 | B31-A | *Δp66/* K04 +*p66* restored to chromosome (Gm^R^) in GCB3214 + pTM61-*strep* (GFP, Sm^R^) | Gent 100 µg/ml Strep 50 µg/ml | This work |  |

**Table S1. *B. burgdorferi* strains used.**

**References**

1. Bono JL, Elias AF, Kupko JJ, III, Stevenson B, Tilly K, et al. (2000) Efficient targeted mutagenesis in *Borrelia burgdorferi*. J Bacteriol 182: 2445-2452.

2. Norman MU, Moriarty TJ, Dresser AR, Millen B, Kubes P, et al. (2008) Molecular mechanisms involved in vascular interactions of the Lyme disease pathogen in a living host. PLoS Pathog 4: e1000169.

3. Kawabata H, Norris SJ, Watanabe H (2004) BBE02 disruption mutants of *Borrelia burgdorferi* B31 have a highly transformable, infectious phenotype. Infect Immun 72: 7147-7154.

4. Moriarty TJ, Norman MU, Colarusso P, Bankhead T, Kubes P, et al. (2008) Real-time high resolution 3D imaging of the lyme disease spirochete adhering to and escaping from the vasculature of a living host. PLoS Pathog 4: e1000090.

5. Elias AF, Stewart PE, Grimm D, Caimano MJ, Eggers CH, et al. (2002) Clonal Polymorphism of *Borrelia burgdorferi* Strain B31 MI: Implications for Mutagenesis in an Infectious Strain Background. Infect Immun 70: 2139-2150.

6. Ristow LC, Miller HE, Padmore LJ, Chettri R, Salzman N, et al. (2012) The β3-integrin ligand of *Borrelia burgdorferi* is critical for infection of mice but not ticks. Mol Microbiol 85: 1105-1118.

7. Ristow LC, Bonde M, Lin Y-P, Sato H, Curtis M, et al. (2015) Integrin binding by *Borrelia burgdorferi* P66 facilitates dissemination but is not required for infectivity. Cellular Microbiology 17: 1021-1036.

8. Labandeira-Rey M, Skare JT (2001) Decreased infectivity in *Borrelia burgdorferi* strain B31 is associated with loss of linear plasmid 25 or 28-1. Infect Immun 69: 446-455.

9. Wu J, Weening EH, Faske JB, Hook M, Skare JT (2011) Invasion of eukaryotic cells by *Borrelia burgdorferi* requires β1 integrins and Src kinase activity. Infect Immun 79: 1338-1348.
